# The impact of location tax incentives on the growth of rural economy: evidence from Ghana

**DOI:** 10.1186/s43093-022-00163-3

**Published:** 2022-11-18

**Authors:** Eric Amankwaah, Nicholas Mensah, Nana Okyir Baidoo

**Affiliations:** 1 Ghana Communication Technology University, PMB 100, Accra North, Ghana; 2 GCB Bank, Kumasi Main, P. O. BOX 852, Kumasi, Ghana; 3 University of Ghana, P.O Box LG 1181, Legon, Accra, Ghana

**Keywords:** Location tax, Incentives, Rural economy, Job creation, Development

## Abstract

Over the last two decades, location tax incentives programs serving as a motivating force have been progressively well known as activities to draw in and/or hold foreign direct investments (FDIs) in economically blighted areas. The study examined the impact of location tax incentives on the growth of the rural economy of Ghana from the period of 1994 to 2018. The data were sourced from Ghana Investment Promotion, UNCTAD, and the World Tax Database. Using ARDL Cointegration and Error Correction Models were estimated to examine the static and dynamic long-run effects as well as the short-run dynamics of the system and the speed of adjustment to the long-run equilibrium. However, FDI is skewed towards the Greater Accra region, Ashanti region, Central region, and Eastern and Western regions notwithstanding the location tax incentives the government has been given since the 1990s. The study shows that there is a positive and statistically significant effect of tax incentives on employment creation and rural development in the long run. It revealed that trade openness and market size have a significant effect on employment creation and rural development. It emerged that there is no correlation between location tax incentives and the regional distribution of FDI in Ghana. It has become a well-established fact that adequate tax holidays are beneficial to businesses and encourage them to make investment decisions. It is therefore recommended that a more extensive mindfulness crusade through workshops, courses, and advertisements is necessary to empower the investors about the accessible business opportunities.

## Introduction

Few countries can remain competitive in today's globalized economy without foreign direct investment. With the potential benefits of technology transfer, expanding business, skill change, and development, it is no surprise that many governments provide location tax incentives [1]. Location tax incentives, according to UNCTAD, are a type of incentive that reduces a party's tax burden to encourage them to invest in specified activities or divisions. These are exceptions to the standard tax system, which may include lower tax rates, tax holidays, accounting methods that account for rapid degradation and acknowledged loss for tax purposes, and tariff reductions or increases on imported products to boost the local market. The goal of location tax incentives is to increase investment prospects in areas where the tax system is perceived as a barrier. They are also used to improve residents' social welfare, such as by providing incentives related to health, education, or saving for future consumption. Furthermore, location tax incentives can be used to discourage specific economic activities, such as farm product overproduction, which creates inflation [2]. Giving incentives has the purpose of improving stock market performance.

Most governments often use location tax incentives to collaborate with the private sector on development projects. Location tax advantages, in particular, are an important part of many governments' economic development strategies. They are used to achieve objectives related to economic growth and job creation, such as balancing economic activity across the country and focusing on high-value enterprises. In addition, the government uses location tax incentives to compete with other states and other countries for the commercial organizations that promise employment and more financial flow.

Proponents argue that tax incentives are necessary to attract businesses and that the costs of these incentives are partially or totally offset by more tax revenues generated by increased economic activity. Opponents argue that location tax incentives are a waste of resources since they spend limited available funds on initiatives that would not have been done without the tax breaks. Several states have requested more detailed reports and evaluations, and the Government Accounting Standards Board has established new rules for monetary declarations that necessitate the inclusion of tax discount reports, a special sort of financial incentive [3, 4].

The choice of international organizations' locations in other jurisdictions was investigated utilizing Dunning’s eclectic or Ownership, Location and Internationalization (OLI) theory [5]. According to Dunning [5], a foreign organization ensures that it has a competitive advantage over other organizations in a specific movement (ownership advantage). The target nation provides benefits that cannot be found elsewhere (location advantage), and the target nation is eager and willing to keep these advantages to itself (internalization advantage) before moving to another nation. This demonstrates that Dunning's eclectic OLI theory is unmistakable in explaining why a company would need to relocate to a jurisdiction different than its own.

According to economic geography theories, different districts of a country are given different levels of transportation, other social and financial infrastructure, communication infrastructure, administrative organizations and business administration convenience, beachfront territories with ports open to global markets, labor markets, and an existing cluster of organizations. Localities that can profit from all or almost all their enhancements are considered affluent, while those that require them are considered less prosperous [6, 7]. The accessibility of these facilities lowers the cost of starting a company's commercial activity. The differences in how these improvements are applied create attractive places and distant districts for FDI in a country, with FDI preferring prosperous/appealing areas over less prosperous/segregated districts. This has a significant impact on job development and accessibility across the country.

The trend has been for people in rural areas that lack FDI and, therefore, job possibilities to migrate to more enticing areas in search of nonexistent jobs. Many governments have used location tax incentives to influence the geographic conveyance of FDI away from preferred locations to discourage people from relocating from remote regions to enticing regions where jobs are scarce. Most of the incentives for limiting are concessions that "reduce the tax burden of companies to encourage them to devote resources to specific activities, locations, or districts" of the host nation [8]. Ordinarily, location incentives take the form of aggregate or partial tax exemptions, tax reductions, accelerated emanation rights, and fare incentives [8]. There is no consensus in the research on their suitability, but governments are increasingly using them as a tool to disperse interests in the country [8, 9].

Historically, investments in Ghana's economy have traditionally been concentrated in four distinct regions: Greater Accra, Ashanti, Central, and West, with metropolitan centers such as Tema, Accra, and Kumasi being the most popular [9, 10]. It should be noted that the four places mentioned above are the country's most well-prepared districts in terms of economic and social infrastructure, skilled labor supply, market dynamism, natural resources, and several other factors. The other six areas with fewer resources are Volta, Eastern, Northern, Upper East, and Upper West. The job possibilities in these less fortunate locations have pushed young people to migrate to more prosperous areas, notably metropolitan centers such as Accra and Kumasi, in pursuit of nonexistent jobs.

The government of Ghana, among other arrangements, makes extensive use of location tax incentives to ensure the geographical distribution of investment and, especially, FDI. Incentives under Law 478 of the Ghana Investment Promotion Center and Law 865 of 2013 are expressed as a tax rebate for investment opportunities for organizations as follows: manufacturing organizations in Accra and Tema benefit from a 25% corporate tax rebate, while organizations in the various provincial capitals benefit from an 18.75% corporate tax rebate.

Corporations headquartered outside of provincial capitals are taxed at a rate of 12.5%. Furthermore, agro-processing companies in Accra and Tema received 20% of the total, while those in other local capitals (excluding Tamale, Bolgatanga, and Wa) received 10%. Those in the North, East, and West receive 0%, while those outside of territorial capitals receive 0%. The motivating force structure was designed to assist manufacturing and agribusiness firms in locating themselves in underserved areas to adapt to economic development and improvement, reduce rural unemployment, and prevent rural–urban migration [11, 12].

Over the last two decades, location tax incentive schemes that serve as motivational drives have been more well-known efforts to attract and/or retain businesses in economically depressed areas. According to McDonald [13], in Britain, more than 40 US states have implemented economic revitalization programs, most often known as Commercial Area Programs (EZ) in the US that provide tax incentives to businesses located in (or near) actual monetary and social problems [14].

Ghana established location tax incentives in the form of a tax reduction for investment in 2000 under the Ghana Investment Promotion Center Act 478 (since substituted by Act 865 of 2013) to ensure the geographical distribution of investments, especially for FDI. Location tax incentives, in particular, are an important part of many countries' economic development plans. They help to achieve and expand economic activity across the country by focusing on perceived high-value firms to boost economic development and job creation.

Despite the government's best efforts to change industrial taxes, there is still a lot of controversy concerning their efficiency across the world. Many proponents argue that industries spurred by incentives (or other changes in tax policy) would have been located in regions where incentives were not used [15], to the point where investment would have occurred without the use of incentives. Tax money has been squandered by local governments. Many opponents of development tax incentives are convinced that this situation occurs as a rule of recruiting, while proponents of tax incentives argue that additional investment would not have happened in the region if the incentives were not in place.

Despite the enormous popularity of tax incentive programs for community development, the available empirical evidence [16–18] on their effectiveness in community development has focused on cross-sectional data. The findings are inconclusive (primarily in the European Union and the United States), and the findings may not adequately address each country's specific problems for policy recommendations that could be easily converted into proposals to the government. The outcomes of the research suggest that location tax incentives have a wide range of strategic application features. This variability is a valuable resource since it stems from strategic decisions that are unlikely to be linked to future economic trends in the target locations. In addition, to the best of my knowledge, only a few empirical studies have been conducted in Ghana. Rousseau and Wachtel [19] propose more research into unique national experiences in order to better understand the influence of location tax incentives on community development. This study presents an empirical investigation into the influence of location tax incentives on community development, with evidence from Ghana.

Even with the immense popularity of tax incentives programs for community development, the available empirical evidence [16–18] on their effectiveness in community development have focused on cross-sectional data, the findings are inconclusive (mainly in Europe Union, United States) that may not adequately address each country's specific problems for policy recommendation that could be effortlessly converted into proposals to refine the future topographically target intervention. The findings from the studies show that location tax incentives indicate incredible heterogeneity in their strategy usage qualities. This heterogeneity is a precious resource since it originates from strategy choices that are in likelihood not related with future economic patterns in the targets regions. Also to the best of my knowledge, only few empirical works have been done in the Ghanaian context. Rousseau and Wachtel [19] therefore call for further studies on country specific experiences to better understand the impact of location tax incentives on community development. This study presents an empirical investigation into the influence of location tax incentives on the growth of rural community, with evidence from Ghana.

## Ghana in context

Ghana’s economy is based on agriculture. Between 1990 and 2018, agriculture's share of employment declined from 55 to 33%. The mix of value added has evolved away from agriculture investment toward services, with extractives playing a significant role. Figure 1.8b illustrates that the value-added share of 'Other Industry' has increased significantly over the last decade, owing mostly to the contribution of Mining and Quarrying, which includes oil and gas production; however, this has not been matched by an increase in the sector's employment share. As a result, the trends of employment and value-added shares by industry have diverged [20].

A period of economic stability began in the early 1990s, ushering in a new age of prosperity, with annual per capita GDP growth averaging 1.9% between 1993 and 2005, fueled mostly by the expansion of the services sector. The restoration to democracy under constitutional authority brought about political stability, which resulted in an economic dividend. In the year 2000, a new administration spearheaded changes in the private sector and macroeconomic management, laying the groundwork for future prosperity [20].

Ghana's accomplishment in reducing poverty during this time has been hailed as outstanding throughout the region. From 2006 onwards, growth grew dramatically. Then, from 2005 and 2019, development accelerated significantly, with annual average GDP per capita growth of 4.1% and aggregate GDP growth of 6.6%. This is much higher than non-high-income Sub-Saharan African nations' average GDP growth of 2%, low-income countries' average GDP growth of 2.6%, and somewhat higher than lower-middle-income countries' average GDP growth of 4.4%. After returning to its 1960s level after 45 years, GDP per capita nearly quadrupled in these 15 years (see Figure 1.2). The transfer of workers from agriculture to services intensified structural transformation, FDI inflows surged fast, and trade openness increased [20].

With the beginning of commercial oil and gas production in 2011, per capita GDP growth reached 11.3%. Price increases for Ghana's key commodity exports, such as gold and cocoa, as well as the start of commercial oil production, all contributed to the previous economic acceleration. Following a high in 2011, GDP per capita growth progressively decreased to −0.1% in 2015, considerably behind the IMF's 2014–2016 growth predictions. A combination of falling commodity prices, energy rationing (owing in part to the impact of a severe drought on hydropower generation) and a massive fiscal crisis in 2013 contributed to the downturn.

**Fig. 1:**
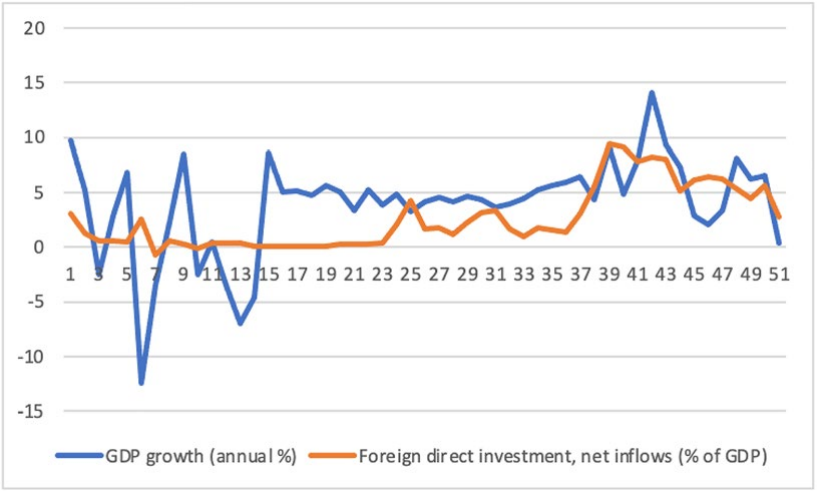
GDP growth (Annual %) and Foreign direct investment. *Source* World Bank [20]

Ghana's economic growth performance increased after 2015, causing hope. After 2015, growth regained again, and the annual GDP per capita growth rate rebounded to 5.8% in 2017. Ghana's economy was one of the fastest growing in Africa in the years leading up to 2020, with GDP per capita increasing by an average of 4.1% per year in 2018–19. Export growth was strong, putting the merchandise trade balance in the black and helping to close the current account deficit. During this time, service exports and FDI inflows rose quickly, and Ghana has been one of the region's top per capita FDI beneficiaries (Fig. 1).

Ghana's job mix has transitioned away from agriculture, especially after 2005, with the fall mostly compensated by services and, more recently, manufacturing. A constantly restrictive monetary policy stance aided in the gradual reduction of inflation. Strong net inflows on the balance of payments resulted in consistent reserve growth. Ghana has made progress in increasing access to power and infrastructure during this time.

### Literature review

Numerous empirical research on the root causes of the issue in Africa have been driven by the continent's rising unemployment and rapid growth. However, the impact of tax incentives on the unemployment issue has not received enough attention. In light of this, Raifu et al. [21] use the real oil prices of Brent and West Texas International along with linear and nonlinear autoregressive distributed lag (NARDL) estimate methods to examine the impact of changes in oil prices on the unemployment rate in Nigeria. According to results from the linear ARDL, fluctuations in oil prices have little to no impact on the unemployment rate. According to the NARDL findings, both a rise and a drop in oil prices have little short-term beneficial effects on unemployment. However, over time, rising oil prices make the unemployment problem worse, while falling prices have little effects. Additionally, we discover proof of a long-term asymmetric link between oil prices and unemployment.

Obiakor et al. [22] study the symmetric and asymmetric consequences of the FDI-growth nexus in Nigeria between 1983 and 2020, taking into account financial crises, economic crises, and COVID-19 pandemics. In contrast, the asymmetric model reveals that parameter estimates have asymmetric impacts on economic development in Nigeria during the current economic crisis. The analysis suggests that FDI inflow/outflow is substantially related with economic growth both in the long and short term. To be more precise, positive shocks to FDI influx or outflow have a large decreasing influence on economic growth whereas negative shocks have a significant accelerating impact.

Similarly, Okere et al. [23] investigate the relationship between trade openness, FDI inflow, and economic growth of Nigeria using time series data from 1982 to 2018. Using the Bayer and Hanck approach to cointegration and the augmented autoregressive distributed lag (AARDL) method. The findings show that (1) the global economic crisis considerably slows down economic growth. (2) The link between trade and FDI-led growth is dampened by the unfavorable interactions between total trade, FDI, and the global financial economic crisis. (3) In contrast to the short term, the long-term effects of FDI influx on the global economic crisis are more severe and significant.

The threshold impact of environmental deterioration on the FDI-poverty nexus in sub-Saharan Africa from 1986 to 2018 is examined by Dada and Akinlo [24]. Panel threshold regression was employed in the study's empirical analysis. The results of threshold regression employing various metrics of environmental degradation and poverty demonstrate that the impact of FDI on reducing poverty is not diminished by environmental deterioration. The study discovered compelling evidence that FDI significantly reduces poverty at the higher level of environmental degradation, except when household final consumption is used as a proxy for poverty, and that FDI has negligible effects on poverty reduction at the higher level of methane emissions and nitrous oxide emissions.

The relationship between foreign assistance and Chad's economic expansion is examined by Kirikkaleli et al. [25] using annual data from 1982 to 2018. The study established connections between the economic indicators using ARDL, FMOLS, and DOLS methodologies. The study then applied the wavelet coherence approach to determine the relationship between economic growth and the independent variables, including its causation. The pattern and behavior of the variables employed, including the various time ranges, are displayed by the wavelet technique, which is one of its distinctive features. We thus investigate the dynamic effects of gross capital creation, foreign assistance, import, and export on Chad's economic development. The outcome of the ARDL long-run estimations shows that foreign aid and gross capital formation have negligible effects on GDP growth. Imports and exports, however, have a positive and considerable influence on GDP growth. The Chadian economy has also been negatively and significantly impacted by the global financial crisis. The results of the ARDL long-run outcomes are supported by the wavelet coherence test results. Therefore, we proposed that significant macroeconomic changes and attempts for economic liberalization will aid in the spread of knowledge, encourage home investment, and facilitate the import of high-tech goods.

## Methods and material

### Research design

A quantitative research strategy was adopted as a way of managing numbers and anything that is measurable efficiently under investigation of phenomena and their links. It is used to answer questions about relationships between measurable components in order to explain, predict, and regulate phenomena [26]. The quantitative research concludes with the confirmation or disapproval of the hypothesis tested. Researchers who utilize the quantitative technique identify one or several elements that they want to employ in their research and continue collecting data related to those aspects.

This study uses an ordinary least squares regression design to look at the association between community development, job creation, and taxes, particularly tax holidays and variable tax rates, in Ghana between 1990 and 2018. An in-depth examination of the connection was conducted as an engaging investigation, with the goal of revealing the historical context of the link between community development, job creation, and taxation, as well as elucidating the goals of the relationship.

## Description of data and sources

The data for the study come from secondary sources. The Ghana Investment Promotion Center (GIPC) provides local and potential yearly business information, as well as location tax incentives from the Ghana Revenue Authority. Trend analysis was used as a tactic in the study. The study adopted this approach in particular when analyzing trends in the geographical delivery of FDI and the creation of jobs from 1990 to 2018. That is, the study looked at the example of FDI placement in the country prior to the implementation of the Localization Incentive Policy and the situation after it was implemented. The study has ramifications for the development and accessibility of work. This study relied on secondary quantitative data. Quantitative data are information that may be expressed numerically and is typically broken down scientifically via the use of factual or econometric methodologies. In terms of quantitative data, this study mostly deals with secondary data, which may be efficiently obtained and reviewed. Panneerselvam [27] said that collecting secondary data increases the expenses, time, and effort required to obtain the information. Data that have recently been created for the maker's first use and for subsequent use by others are referred to as "secondary data."

## Model specification

The study used multiple regressions to determine the effects of tax incentives (tax exemption and tax exemption) on community development and to determine if job creation has a different effect. The specification of the model is based on the theory of neoclassical investment, which predicts a flow of development and increased job creation following the provision of tax incentives to decrease the tax burden. This theory aligns investment with marginal performance after capital taxation. Taxes influence economic development and job creation through rates of return on capital. According to the theoretical framework and the previous work of [28] and [29], this study used incentive effects on community development and job creation. The following function is the fundamental empirical model we propose for modelling Tax incentives and community development & job creation, which are indicated by TINit and Yit, respectively.

1 $$Y_{it} = A_{it} + {\text{TIN}}_{it} + \varepsilon_{it}$$

where the index *i* = 1, *t* = 1, 2…., *T* denotes time, *Y* = community development and Job creation, stochastic error term, *A* = other variables.

The model was modified to take into account the peculiarities of the country and the theories. Community development and job creation are the dependent variables that measure the inflow of development activities in the community *i* at time *t*, which is defined as the dollar value of the development projects. Therefore, for this analysis, the study estimates the following long-run models:

2 $${\text{CD}}_{it} = \beta_{0} + \beta_{1} {\text{TIN}}_{t} + \beta_{2} {\text{MS}}_{t} + \beta_{1} {\text{TOP}}_{t} + \varepsilon_{it}$$

To test whether the appropriate tax incentive policy actually increases employment, we propose the second model.

3 $${\text{JBC}}_{it} = \beta_{0} + \beta_{1} {\text{TIN}}_{t} + \beta_{2} {\text{MS}}_{t} + \beta_{3} {\text{TOP}}_{t} + \varepsilon_{it}$$

where CD is community development, and JBC is job creation. Other variables TNC = tax incentives, MS = market size and TOP = trade openness. *ε* is stochastic error term, and *β*0 is an intercept of the sector (It basically states that the value of the dependent variable will equal the constant term if all of the explanatory variables in the model are zero at a certain moment), *β*s are the coefficients of the variables (When the predictor is changed by one unit, the coefficient value shows the average change in the response).

To estimate the short-run model for this study, it is necessary to estimate the error correction model. Thus, the error correction model result demonstrates the speed of adjustment back to the long-run equilibrium after a disturbance [30]. Thus, the expected short-run community development and job creation are indicated by Eqs. (4) and (5) respectively.

4 $$\Delta {\text{CD}}_{t} = \beta_{0} + \sum\limits_{i = 1}^{p} {\theta \Delta {\text{TIN}}_{t - i} } + \sum\limits_{i = 1}^{q} {\beta_{1} \Delta {\text{TIN}}_{t - i} } \sum\limits_{i = 1}^{r} {\beta_{2} \Delta {\text{TIN}}_{t - i} } \sum\limits_{i = 1}^{s} {\beta_{3} \Delta {\text{TIN}}_{t - i} } + \psi {\text{ECT}}_{t - 1}$$

5 $$\begin{gathered} \Delta {\text{JBC}}_{t} = \beta_{0} + \sum\limits_{i = 1}^{p} {\theta \Delta {\text{TIN}}_{t - i} } + \sum\limits_{i = 1}^{q} {\beta_{1} \Delta {\text{TIN}}_{t - i} } + \sum\limits_{i = 1}^{r} {\beta_{2} \Delta {\text{TIN}}_{t - i} } + \sum\limits_{i = 1}^{s} {\beta_{3} \Delta {\text{TIN}}_{t - i} } + \psi {\text{ECT}}_{t - 1} \hfill \\ \quad \quad \quad \quad \quad + V_{t} \hfill \\ \end{gathered}$$

where $$\Delta$$ is difference operator and $${\text{ECT}}_{t - 1}$$ is error correction term lagged one period. The coefficients $$\beta_{1}$$*,* $$\beta_{2}$$$$\beta_{3}$$ are the elasticities of the respective variables, with $$\psi$$ showing the speed of adjustment, $$\beta_{0}$$ is the drift component, *t* denotes time and $$\nu_{t}$$ is the stochastic error term [30]. The coefficient of the lagged error correction term is expected to be negative and statistically significant to further confirm the existence of a cointegrating relationship.

## Descriptions of variables

### Dependent variables

#### Community development

Community development is a measure of a number of FDI projects (as a proxy of the rural economy) that can be seen as a way to deal with the country's improvement. Community development focuses on bringing people together within a geographical area. Several studies have found that tax incentives have a major impact on community development [2, 16–18].

#### Employment (job) creation

Employment creation is hard to assess because it is hard to quantify. Employment creation is measured by the expected employment created by FDI projects from 2001 to 2018. The famous brilliant egg of employment creation arrangements is the "net new occupation"—the activity that is created without uprooting some other economic activities. While it is simple to determine whether another activity has been created at the macroeconomic level by looking at total data from the Bureau of Labor Statistics, it is extremely difficult to determine whether (1) the occupations created did not only displace occupations in different areas or segments, and (2) if the occupations were created in response to a specific arrangement [31]. This issue arises often throughout this study; the potential mechanism for how an arrangement creates jobs may be undeniably known, but evidence indicating that it really produces net new jobs is conflicted, best case scenario, or, more commonly, almost nonexistent. The investments cut across all sphere of economic activities from service, manufacturing and mining sector.

### Independent variables

#### Tax incentives

Tax Incentive is the explanatory variable with emphasis on tax incentives, taxes related to tax exemptions, and tax holidays from customs duties that are the most popular incentives in Ghana. Given the difficulty of measuring fiscal incentives, this study uses a fictitious variable to show the presence and absence of fiscal incentives in the sectors considered [1]. It is assumed that the tax incentives per annum are a measure of location tax incentives. As a result, a positive relationship is anticipated.

Other control variables that affect community development and the creation of employment are those that control the market size and the openness of the economy. Although many variables have been proposed in various kinds of literature as determinants of FDI, it is not possible to include them all. As a result, we chose a few based on previous studies specific to the country, the strength of the variable, and the availability of data.

#### Market size

The market size is a proxy of GDP, which must be positively related to community development and job creation. The greater the market size, the greater the community development in terms of projects and the availability of jobs [32, 33].

#### Trade openness

Trade openness measures the opening of the economy and its integration into the global economy. It is captured by the ratio of imports to exports in relation to GDP. A higher relationship represents more openness [34, 35]. A positive relationship with incentives is also expected. This means that the greater the relationship, the more open the economy is and the greater the inflows of FDI.

#### Estimation technique

The influence of tax incentives on community development and employment creation was studied using the maximum likelihood estimate (MLE) approach. The MLE approach is a method for estimating population parameters (such as mean and variance) from data that picks as estimates those parameter values that have the best chance of matching the observed data. The maximum likelihood estimation process may be used for a broad range of models, and it typically produces estimators with excellent asymptotic characteristics [36].

The order of integration and time series characteristics of the data used in the Augmented Dickey–Fuller (ADF) and Phillip–Perron (PP) models were examined first. Second, the study uses the Autoregressive Distributed Lag (ARDL) technique to cointegration to examine the short-and long-run correlations between the variables [37]. The ARDL technique to cointegration is said to be the single equation equivalence of Philips-(1990) Hansen's completely modified ordinary least squares procedure's maximum likelihood approach [37]. The ARDL model's stability and diagnostic test statistics, as well as granger causality analysis, were also analyzed to confirm the model's reliability and goodness of fit.

## Results and discussions

### The degree of regional imbalance in the distribution of FDIs projects

**Fig. 2:**
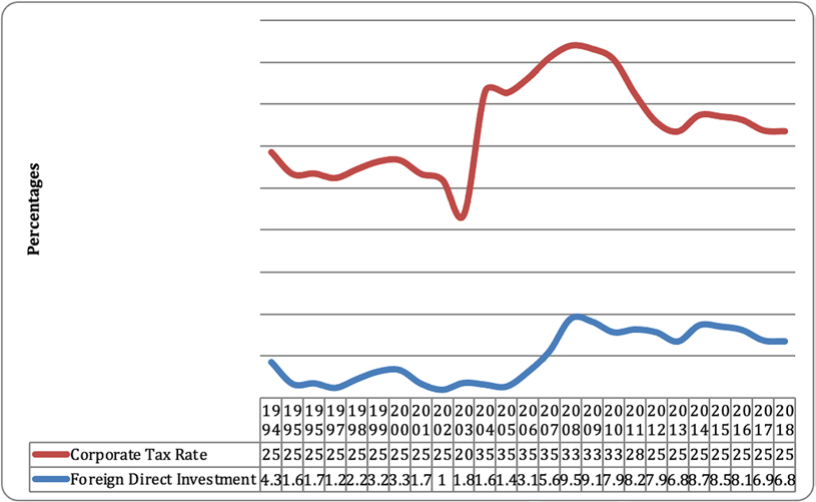
FDIs and corporate rates in Ghana. *Source* UNCTAD 2018, World Tax Database 2018

Ghana's tax system has been in place since the 1990s, and it is updated on a regular basis in order to encourage foreign direct investment. The study looked at the degree of regional imbalance in the distribution of FDI projects using trend analysis. The analysis is done as follows:

Figure 2 demonstrates the consistent growth of total foreign investment from the period of 1994 to 2018. The total foreign direct investment ranges from 1.73 to 9.5%. After 2005, the growth of FDIs detonated. Total FDIs increased by 3.12% in 2006, a 1.39% increase. Total foreign direct investment increased by 9.6% in 2008.As a result; it began to fall from 2009 to 2018, falling from 9.13 to 6.77% from 2009 to 2018.

Figure 2 also shows that Ghana has demonstrated an expansion in total FDI. We can accept that these progressions have had a constructive outcome. Corporate tax rates have a likely impact on FDIs growth. The corporate tax rate was reduced from 2002 to 2006. Each time the corporate tax rate falls, the total FDIs expands. This implies that there is a link between corporate tax rates and FDIs. A further analysis is done in Table 1 to examine the degree of regional distribution of FDI projects.

**Table 1:** Regional distribution of FDI Projects

Region	Totals	Percentages
Upper West	4	0.07
Upper East	8	0.14
Brong Ahafo	43	0.78
Northern	54	0.97
Volta	64	1.15
Eastern	127	2.29
Central	119	2.15
Western	231	4.17
Ashanti	318	5.74
Greater Accra	4576	82.54
Total	5544	100.00

*Source* GIPC, 2018

**Fig. 3:**
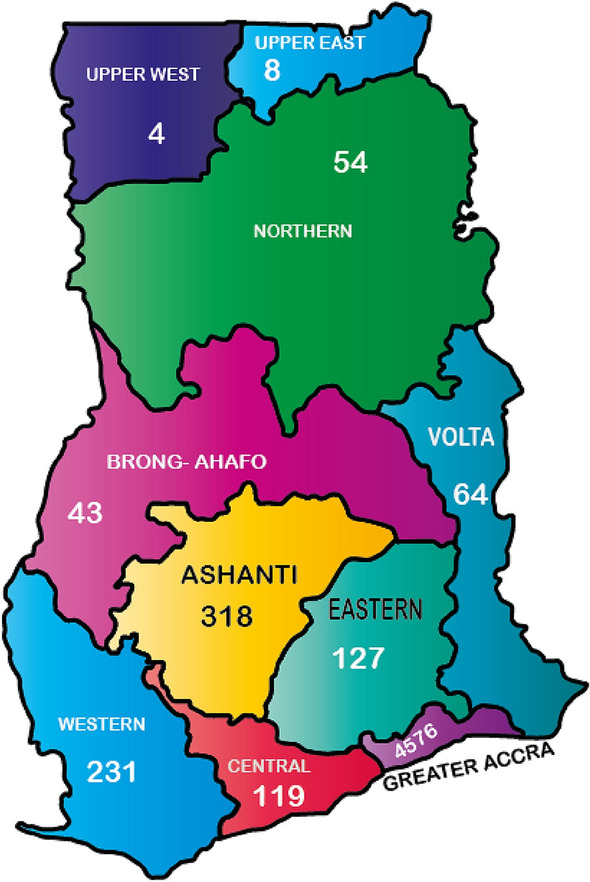
Map showing regional distribution of FDI projects. *Source* GIPC, 2018 & Authors’ Construct, 2022

The study used trend analysis to examine the first objective of the study. Table 1 and Fig. 3 demonstrate that the FDI regional distribution of FDI is skewed towards the Greater Accra region, the Ashanti region, Western, Central and Eastern region of Ghana. Specifically, from the period of September 1994 to 2018Q4, the Greater Accra recorded 4576 of the total 5544 FDIs received in Ghana representing 82.54% of FDI inflows. The Greater Accra region is followed by the Ashanti region with 318 FDIs representing 5.74% of the total FDIs, the Western region with 231 FDI indicating or 4.17% of the total FDIs, and the Center region with 119 FDIs thus 2.15% of the total FDI inflows and Eastern Region with 127 FDIs indicating 2.29% of the total FDIs inflows in the country. The five regions together got 5371 FDI from the total of 5544 FDIs enjoyed in the country. In percentage terms, the five regions together represented 96.89% of the entire total FDI enjoyed during the study period. The other regions received the remaining 173 (3.11%) of FDI received by the nation were shared among the five outstanding regions: Volta, North, Brong Ahafo, Upper East and Upper West. Specifically, the Volta region received 54 (1.15% FDIs), Northern received 54(0.97% FDIs), Brong Ahafo received 43(0.78% FDIs), while Upper East and Upper West received just 8(0.14%) and 4(0.07% FDIs), respectively. The outcomes are appeared in Table 1. It is noted that FDIs activities ought to generate potential employments. This implies that if such activities are enrolled in a region, more occupations are bound to be created in that region. Actually, the Greater Accra region, the most skilled region that hosts the majority of these FDI ventures, has a larger number of employments than less talented regions, like the Upper East and the Upper West.

### Descriptive statistics

This section of the study provides the descriptive statistics of the selected variables. The descriptive statistics include the mean, median, maximum, minimum, standard deviation, skewness, kurtosis, sum, and number of observations. Table 2 illustrates extensively these statistics.

Table 2 demonstrates that all variables have positive average values (mean and median). This is usual considering the arrangement included. Then again, the minimum deviation of the variable of their means, as appeared by the standard deviation, gives an indication of moderate growth (fluctuation) of these variables amid the considered period. Regarding skewness, all variables (except employment creation) are positively skewed. The Jarque–Bera measurement that demonstrates the null hypothesis that all the series are gotten from a normally distributed random process can not be rejected for market size implying that they are normally distributed. But community development, tax incentives, and trade openness are not normally distributed. As a result, the ARDL approach was employed for the analysis since it assumes a normal distribution not for all the series or variables.

**Table 2:** Summary statistics of the variables

Statistics	CD	JBC	INC	TOP	GDP
Mean	3.86e-16	2.75e-15	27.2	56.0531	5.319191
Median	0.027858	0.094470	15.73125	46.0200	4.651426
Maximum	0.293941	1.162529	35	116.0600	14.04712
Minimum	−0.318440	−1.390685	20	6.1900	2.282918
Std. Dev	0.162685	0.589889	4.582576	29.7858	2.207131
Skewness	0.044368	−0.400252	0.0255	0.1953	1.886337
Kurtosis	2.277970	2.980920	0.8232	1.9449	7.464251
Jarque–Bera	2.529202	0.561024	4.9800	2.3734	56.93767
Probability	0.767512	0.755397	0.0831	0.3052	0.00000
Sum	1113.810	520.9000	680.000	2522.390	212.7677
Observations	25	25	25	25	25

*Source* computed using Eviews 10.0 Package

### Results of the unit root test

Although the ARDL cointegration approach can be used independently of the order of integration, it is essential to verify the unit root test to determine if the variable are not integrated into an order higher than one with a specific one in order to extricate the results from spurious regression. This is on the grounds that the computed F-statistics gave by Pesaran et al. [38] won't be legitimate within the presence of I(2) variables.

The Augmented Dickey–Fuller (ADF) and Phillips and Perron (PP) tests were linked to all variables in the levels and first difference to formally build up their order of integration. The Schwarz–Bayesian Criterion (SBC) and the Akaike Information Criterion (AIC) were utilized to decide the ideal number of lags incorporated into the test. The study used the P value to settle on the unit root decision (i.e., rejection or acceptance of the null hypothesis that the series contains a unit root) that reached a comparable conclusion with critical values.

In Table 3, the invalid theory of the root unit for all variables can not be rejected at levels. This implies not all variables are not stationary at the level since their p value for the ADF and PP tests are not significant at all conventional level of significance.

**Table 3:** Unit root test for the order of integration (ADF and Philips Perron) at Levels with (intercept and trend)

Variable	ADF Stats	*P* Value	Lag	PP Stats	*P* Value	[BW]
CD	−3.17085	0.1059	[2]	1.6609	0.9995	[3]
JBC	−1.921128	0.6240	[1]	−4.712977	0.0027	[3]
INC	−4.399273	0.0062	[0]	−4.400589	0.0062	[2]
TOP	−3.101025	0.1203	[1]	−3.113519	0.1174	[3]
MS	−3.870085	0.0231	[0]	−3.910709	0.0209	[2]

*Source* Computed using Eviews 9.0 Package

Table 4 shows that, at first difference, all the variables are stationary and we reject the null hypothesis of the existence of unit root. We reject the null hypothesis of the existence of unit root in D(CD), D(JBC), D(INC), D(TOP) and D(TOP) and at the 1% level of significance. From the above analysis, one can therefore conclude that all variables are integrated of order one I(1) and in order to avoid spurious regression the first difference of all the variables must be employed in the estimation of the short-run equation. The results demonstrates that community development, employment creation, tax incentives, market size and trade openness are not stationary at levels. This is on the grounds that the *P* value ​​of the ADF statistic are not statistically significant. However, when these variables are differenced for the first time, they end up noticeably stationary. Therefore, the null hypothesis of the existence of unit root (non-stationary) at the significance levels of 1% are rejected.

**Table 4:** Unit Root Test For Order of integration: (ADF and PP): at first difference with (intercept and trend)

VARS	ADF STATS	*P* Value	*OI* LAG	PP STATS	*P* Value	*OI* BW
DCD	−10.4876	(0.0000)***	*I(1)* [0]	−10.9737	(0.000)***	*I(1)* [1]
DJBC	−12.0446	(0.0000)***	*I(1)* [1]	−12.1699	(0.000)***	*I(1)* [2]
DINC	−6.56452	(0.000)***	*I(1)* [0]	−13.8240	(0.000)***	*I(1)* [3]
DTOP	−7.19665	0.0000	*I(1)* [1]	−15.5245	(0.0000)	*I(1)* [2]
DMS	−7.52739	(0.000)***	*I*(1) [0]	−10.4504	(0.000)***	*I*(0) [8]

*Source* Computed using Eviews 9.0 Package

IO represents order of integration and D denotes first difference. ***, ** and * represent significance at the 1, 5 and 10% levels respectively

From Tables 3 and 4, the null hypothesis of the presence of unit roots cannot be rejected for community development, employment creation, tax incentives, market size and trade openness because the *P* values of both the ADF and the PP statistic are not significant at all the conventional levels of significance.

### Bounds test for cointegration

The presence of long-run relationships is tested using the bounds test for cointegration. Cointegration test enabled the verification of existence of both long-run and short-run relationships among the variables used for the analysis. Pesaran and Shin [37] argued that performing a cointegration test allows researchers to establish the existence of an imbalance in several equations or not. According to Charemza and Deadman [39], the presence of a cointegration relationship between variables implies the existence of adjustment mechanisms that prevent errors in the long-term relationship from increasing.

The implication is that whenever two or more variables are cointegrated, then theoretically, there exists an error correction mechanism. The error correction term is the lagged residual of the cointegration regression. It determines the speed of adjustment to long-run equilibrium. For this reason, a negative and significant coefficient of the error term is expected. According to Pesaran et al. [38], the lower bound critical value assumes that the explanatory variables are integrated of order zero or I(0), while that of the upper bound assumes that the explanatory variables are integrated of order one or I(1).

Table 5 illustrates the results of the bound test. From the table, the F-statistic for the model 1 with CD as the dependent variable is 6.875451*** and model 2 with JBC as the dependent variable is 7.767313. It surpasses as far as possible the upper critical bound at one percent level of significance. This implies the invalid speculation of non-cointegration between the variables of the condition is rejected. This proposes a long-term connection between CD and its independent variables as well as a long-term relationship between JBC and its independent variables. The presence of a cointegration between return on equity and the independent variables of the model means that an error correction exists; thus, the investigation permitted to assess the long-run coefficients and the dynamic parameters for the short run.

**Table 5:** Results of bounds test for the existence of cointegration

Model	Dependent variable		Function		F-statistic	Cointegration status
1	CD		F(CD/INC, MS, TOP		6.875451***	Cointegrated
2	JBC		F(JBC/INC, MS, TOP		7.767313***	Cointegrated
*Asymptotic Critical Value*						
Model 1	1%		5%		10%	
	I (0)	I (1)	I (0)	I (1)	I (0)	I (1)
	4.29	5.61	3.23	4.35	2.72	3.77
*Asymptotic Critical Value*						
Model 2	1%		5%		10%	
	I (0)	I (1)	I (0)	I (1)	I (0)	I (1)
	4.29	5.61	3.23	4.35	2.72	3.77

*Source* Estimated using Microfit 5.1 package

*, **, and *** shows 10, 5 and 1% levels of significance respectively

### Analysis of the long-run results between tax incentives and community development

The government encourages FDI in specific business development projects with tax relief on capital and gains if the government thinks the project is beneficial for Ghana. To promote rural development, the government offers tax incentives in the form of relief from stamp duty and full tax exemption from corporate income tax, dividend tax, and capital gains tax. This session presents the results and discussion of the long-run connection between community development and tax incentives.

Since the results of the cointegration analysis demonstrates the presence of a long-run connection between dependent variable (community development) and the independent variables, the study continued to assess the long-run effect of the independent variables on community development utilizing the ARDL approach. The assessments of the selected ARDL demonstrate in light of the Schwarz–Bayesian Criterion-ARDL(1,0,0,0,2,0) is displayed in Table 5.

Table 6 demonstrates that the impact of tax incentives on community development, contingent upon the proxies used to gauge community development. When the FDI projects is utilized as proxy of community development, the coefficient of tax incentives is positive and statistically significant at 5% significance level. In particular, 1% expansion in tax incentives will lead to 50.3% increase in community development over the long run. This confirms expectation of the study. The magnitude of the coefficient suggesting that strong tax incentive environment can also weigh on the foreign investors as it will motivate them to increase their investment portfolios. Taxes are used to empower investment and invigorate enterprises. The need for economic segments to have an incentive to improve purchasing power and reduce costs [40]. The implication is that the more such projects are recorded in a region, the higher the probability that more development will be enjoyed in that region. This may probably be that sufficient tax incentives enhance growth and the economy. Further, incentives are high motivators for the establishment of business enterprises. It implies that where incentives are not present, it will not only demotivate people, but it can also lead to the closure of already existing industries, as such incentives should be present so as to keep motivating people and business owners to establish and expand their enterprises. Several studies have found that tax breaks have a significant impact on community development [2, 16–18]. The findings also support El Ha and Zenjari, [41] assertion that while taxation is not the most vital determinant of investment, it greatly affects its intensity and net gainfulness. This finding is also contrary to Fahmi's [42] findings that tax holidays, the fundamental autonomous variable directed, were not significant in pulling in FDI inflows. The study noted that tax holidays can never adjust for a lacking framework, economic and political shakiness, and terrible government strategies. Similarly, Stapper [43] found that the high corporate tax rate does not influence the investment choice of foreign direct investments. Also Šimović and Zaja [44] found that tax incentives are used to draw in investment and develop rural communities.

**Table 6:** Estimated long-run coefficients using the ARDL approach for community development model (Model 1)

Variables	Coefficient	Standard Error	*T*-statistic	*P* Value
C	0.010130	0.092923	0.109013	0.9163
INC	0.502997	0.347191	1.446763	0.0503**
TOP	−0.280889	0.818087	−2.565489	0.0375**
MS	2.098792	1.082450	2.36067	0.0373**

*Source* Computed using Microfit 5.1

*, **, and *** show 10, 5 and 1% levels of significance respectively

Table 6 also indicates that there is a negative and statistically significant relationship between trade openness and rural development in the long run. All other being equal, an increase in trade openness will result in an decrease in rural development of 28.1% over the long-run period. The opening of the economy measures the country's trade openness and its integration into the global economy. In the long run, as they are compelled to compete in the same market as larger economies or nations, trade liberalization can be a threat to emerging nations or economies. This dilemma has the potential to suffocate existing local companies or cause newly generated industries to fail thereby reducing rural development. This support Etim et al.'s [45] ([34, 35]) findings.

Table 6 illustrates that the coefficient of market size is positive and statistically significant to rural development. All other things being equal, an increase in market size will lead to a total increase in rural development in the long run. More importantly, a percentage change in market size will lead to a 209.8% increase in rural development. The higher the market size, the greater the community development in terms of projects and availability of jobs [32, 33]. As the Ghanaian market turns out to be more competitive, industrial motivating forces can be successful on the off chance that they support modern activities with high potential for included worth. The significant role of tax incentives in the economic development of any nation can't be exaggerated. This is on the grounds that it is the premise of a strong reason for the development and long-term improvement of the industrial sector. This finding is in tandem with Etim et al.'s [45] findings that the extent of the market (GDP), the opening, and the conversion scale affected the inflow of FDI, while the political risk was negative.

### Analysis of short-run results for community development model

This session presents the results and discussion of the short-run relationship between community development and tax incentives. The short run is defined as the period one year and below. The Error Correction Model (ECM) gives the methods for accommodating the short-run behavior of an economic variable with its long-run behavior. The presence of cointegration connections among the variables requires the estimation of ECM to comprehend the short-run dynamic behavior of the bank profitability model. The model uses the first difference of the variables (capture short-run changes). The analysis tests the null hypothesis of no short-run relationship between community development and tax incentives as against the alternative hypothesis of a short-run relationship between community development and tax incentives.

The coefficient of the ECM, as shown in Table 7, reveals how quickly the variables converge to equilibrium after a shock and has a statistically significant coefficient with a negative sign, as predicted, in the model at the 5% significant level. This confirms that the variables in the model have a short-run relationship. The span of the coefficient on the error correction term (ECT) in the model shows that about 10.3% of the disequilibrium in community development is driven by past years' shocks, and that this year's disequilibrium converges back to the long-run equilibrium. The negative sign indicates that any short-term shock will be compensated for in the long run. According to Acheampong [46], the bigger the absolute size of the error correction component, the faster the convergence to equilibrium. However, the size of the coefficients in this analysis shows that long-run changes are adjusted at a modest rate. A extremely substantial error correction term, according to Bannerjee and Mestre [47], further demonstrates the presence of a long-run connection. When shocked in the short term, the variables in the model exhibit proof of immediate reaction to equilibrium, according to the research.

**Table 7:** Short-run estimates using the ARDL approach (Community development)

Variables	Coefficient	Standard error	*T*-statistic	*P* value
dC	17.9413	36.52344	−0.491227	0.6383
dCD (−1)	0.663194	0.307443	2.457126	0.0379**
dCD (−2)	1.308740	0.309101	4.234026	0.0039**
dINC (−1)	0.659027	0.298861	2.405130	0.0333**
dINC (−2)	2.185666	0.385019	5.676770	0.0008***
dTOP (−1)	1.414981	0.263505	5.369839	0.0010***
dTOP (−2)	4.514618	1.752335	2.576344	0.0367**
dMS (−1)	−2.642398	0.903544	−2.924481	0.0222**
dMS (−2)	−2.868649	1.114105	−2.5748471	0.0367**
ECM (−1)	−0.10301	0.0495	−2.1813	0.035**
R-squared	0.980565			
Adjusted R-squared	0.902827			
F-statistic	12.61362			
Prob (F-statistics)	0.000994			
Durbin-Watson statistic	2.167253			

*Source* Computed using Microfit 5.1

*, **, and *** shows 10, 5 and 1% levels of significance respectively

The F-statistic value shows that tax incentives, market size, and trade openness have a substantial impact on community development (as measured by FDI projects) across the research period. The R-squared (0.980565) estimation in the model shows that the independent variables in the study explain around 98.1% of changes in community development in the short run. The DW-statistics estimate of 2.167253 confirms that there is no serial correlation.

Consistent to the results of the long run, the coefficient of the tax incentives is positive and statistically significant at 5% significant level. Hence, 1% expansion in tax incentives will lead to 66.3% increase in community development, compared to 50.3% over the long run. This shows that the tax incentives is efficient at a high percentage given its strategy of motivating investors to invests in areas that promote community development in the short run and long run. This contradicts to Abdulrahman and Olumide [48] findings.

Contrary to the long-run results, the coefficient of trade openness is positive and statistically significant at 5% significance level in the short run. In this way, a 1% expansion in trade openness has the capability of reduce community development by around 141.5%. The impact of trade openness on community development appears to be more extreme in the short run (141.5%) than over the long run (28.1%). This implies, utilizing trade openness to enhance community development is more viable over the short-term period than in the long run in developing countries. In the short term, integration with global commerce with sources of innovation is facilitated through trade, which boosts FDI gains. Trade openness encourages economies to grow output, resulting in higher returns to scale and economics of specialization [48]. This is in tandem with the findings of Abdulrahman and Olumide [48]

Finally, the coefficient of market size (GDP) is positive and statistically significant, which confirms to the positive relationship acquired over the long run. This implies, over the short run there is a positive connection between GDP and community development in both short and long term. The probability of firm exist increase as the market size increases resulting in an increase in investment projects which intend improve development. This finding concurs with discoveries of Kondo & Okubo [49]

### Model diagnostics and stability tests

The evaluated properties of a time series data, according to Hansen [20], may change with time. As a result, it is critical to do parameter tests to look for model misspecification that might arise as a result of shaky parameters, allowing for a more accurate bias assessment. Tables 8 show the results of the model diagnostics and goodness of fit tests.

**Table 8:** Model goodness of fit and model diagnostic tests

Test Statistics	LM Version		F Version	
A: Serial Correlation	CHSQ (1) =	15.08348 (.005)	F (1, 24)	.1.802820 [.2573]
B: Functional Form	CHSQ (1) =	.46429 (.307)	F (1, 24)	.20263 [.656]
C: Normality	CHSQ (2) =	27.0231 (.000)	Not applicable	
*D: Heteroscedasticity	CHSQ (1) =	.27887 [.597]	F (1, 24)	.16737 [.708]

*Source* Computed using Microfit 5.1

Also, Table 8 shows that the errors are normally distributed and the model passes the Ramsey’s RESET for correct specification of the model as well as the white heteroskedasticity test.

**Fig. 4:**
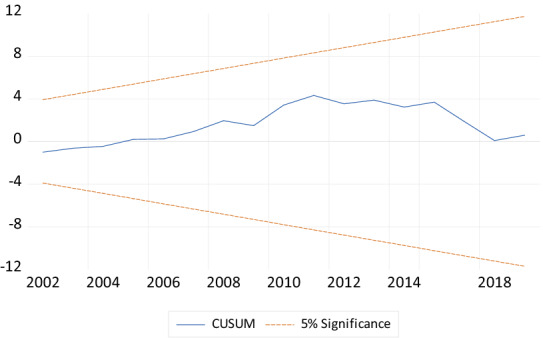
Plot of cumulative sum of recursive. *Source* Generated by the author using Microfit 4.1

Finally, to check the stability of the coefficients of the model, the study employed the CUSUM and CUSUMSQ of recursive residuals stability tests as recommended by Pesaran and Pesaran [50]. According to Bahmani-Oskooee [51], the null hypothesis for this test is that the coefficient vector is the same in every period. The CUSUM and CUSUMSQ of recursive residual stability test in Figs. 3 and 4 indicate that all the coefficients of the estimated model are stable over the study period since they are within the 5% critical bounds.

**Fig. 5:**
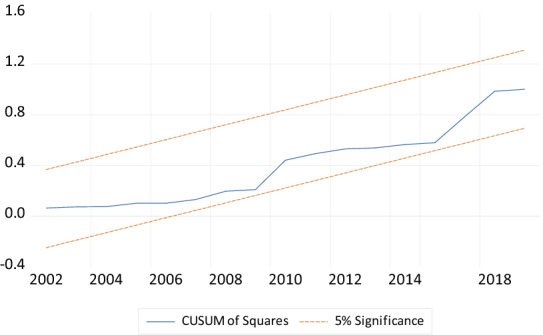
Plot of cumulative sum of squares of recursive residuals. *Source* Generated by the author using Microfit 4.1

From Figs. 4 and 5, the variable on the vertical axis is residuals whiles the variable on the horizontal axis is years. The variable on the vertical axis is residuals whiles the variable on the horizontal axis is years. The CUSUM and CUSUMSQ graph indicates that the coefficients in the models are stable over the study period.

### The effectiveness of location tax incentives in job creation

This section analyses the effectiveness of tax incentives on employment creation. Under the Ghana Investment Promotion Center Act of 1994, the agriculture and manufacturing sectors that fundamentally used local raw materials or engaged in the production of agricultural equipment, building and construction, tourism and mining enjoy some incentives in terms of tax exemption from customs import duties on plants and machinery, reduced corporate income tax rates, and other incentives with the view of creating jobs. It noted that FDI activities ought to generate potential employment. The implication is that if such ventures are received in an area, more occupations are bound to be created in that region.

The study compares the null hypothesis of no long-run relationship between job creation and tax incentives with the alternative hypothesis of a long-run relationship between job creation and tax incentives. The study continued to assess the long-run effect of the independent variables on employment creation using the ARDL approach after the results of the cointegration analysis revealed the presence of a long-run connection between the dependent variable (employment creation) and the independent variables. Table 5 shows the results of the selected ARDL's evaluations in light of the Schwarz–Bayesian Criterion-ARDL(1,0,0,0,2,0).

Table 9 also shows that there exists a positive and statistically significant relationship between tax incentives and employment creation. All other things being equal, an increase in tax incentives will lead to a total increase in employment creation. More importantly, a percentage change in tax incentives will lead to a 57.9% increase in employment creation. Therefore, the null hypothesis that tax incentives have no effect on employment creation is rejected. As indicated by Philip [52], tax incentives are "a deliberate decrease (or aggregate disposal) of tax commitments conceded by the government to empower specific economic units (for instance, organizations must act in an attractive way (for instance, contribute more), deliver more, utilize more, save more, devour less, import less, contaminate less, and so forth. It is therefore necessary for a country to improve employment by supporting private businesses. One reason for encouraging FDIs is that investment activities will encourage them to create business opportunities and jobs for Ghanaians.

**Table 9:** Estimated long-run coefficients using the ARDL approach for job creation

Variables	Coefficient	Standard error	*T* statistic	*P* value
C	0.813477	0.136122	−5.976089	0.0001***
INC	0.578694	0.249347	2.320842	0.0405**
MS	−0.813477	0.183158	−4.441396	0.0010***
TOP	2.285181	0.652558	3.501882	0.0050**

*Source* Computed using Microfit 5.1

*, **, and *** show 10, 5 and 1% levels of significance respectively

The coefficient of trade openness has a positive and statistical effect on employment creation. More importantly, all other things being equal, an increase in trade openness will result in 81.3% increase in job creation. This means that the greater the relationship, the more open the economy is and the greater the inflows of FDI and job creation are. This conforms to Amanuel's [39] findings that the level of transparency and expansion in Ethiopia has significantly affected foreign direct investment flows in Ethiopia.

Finally, market size (measured as GDP) is negative and statistically significant to employment creation. The implication is that Job creation is aided by economic growth. Growth must also boost the productive capacity of sectors that have the ability to absorb significant amounts of labor (for jobs). Martins [53] concludes that economic development was unable to produce adequate productive employment in Ghana. This finding contradicts with Etim et al. [45] finding that market size (GDP), receptiveness and the size of transformation affected FDI inflows. Similarly, Kudaisi [40] found that market size has significant effect on employment creation. Hussain and Kabibi [54] noted the extent of the market is the most essential factor of foreign direct investment in developing nations.

### Analysis of short-run results

The results and discussion of the short-term link between tax incentives and employment creation are presented in this session. The term "short run" refers to time periods of one year or less. The error correction term, according to Bahmani-Oskooee [52], is a more successful way for building up cointegration to some extent. The Error Correction Model (ECM) describes how to reconcile an economic variable's short-run behavior with its long-run behavior. The presence of cointegration relationships among the variables necessitates the estimate of ECM in order to appreciate the bank profitability model's short-run dynamic behavior. The initial difference between the variables is used in the model (capture short-run changes).

From Table 10, the coefficient of the ECM indicates how fast the variables converge to equilibrium following a shock and it has a statistically significant coefficient with a negative sign, as expected, in the model at 5% significant level. This affirms the presence of short-run relationship among the variables in the model. In the model, the span of the coefficient on the error correction term (ECT) demonstrates that around 30.2% of the disequilibrium in employment creation is caused by earlier years' shocks converges back to the long-run equilibrium in the present year. The negative sign implies that any shock that occurs in the short run will be corrected in the long run. Acheampong [55] maintains that the larger the error correction term in absolute value, the faster the convergence to equilibrium. However, the magnitude of the coefficients in this study suggests that the speed of adjusting to long-run changes is moderate. Bannerjee and Mestre [46] noted that a highly significant error correction term further confirms the existence of a long-run relationship. The study indicates that the variables in the model show confirmation of direct reaction to equilibrium when shocked in the short run.

**Table 10:** Short-run estimates using the ARDL approach

Variables	Coefficient	Standard error	*T* statistic	*P* value
dC	0.059825	0.175347	0.341179	0.6142
dJBC	−0.578694	0.177289	−3.264122	0.0075***
dJBC (−1)	0.433413	0.26896	2.559866	0.0376**
dINC (−1)	0.607399	0.220767	2.751312	0.0285**
dTOP	−0.223510	0.122293	−1.827652	0.0948*
dTOP (−1)	−0.813477	0.385019	5.676770	0.0008***
dTOP (−2)	−2.863171	0.0031	2.4773	0.053*
dMS (−1)	1.747147	0.902413	1.936082	0.0941
dMS (−3)	2.041561	0.654200	3.120698	0.0168**
ECM (−1)	−0.302417	0.080571	−3.753417	0.0032**
R-squared	0.809600			
Adjusted R-squared	0.728000			
F-statistic	9.921568			
Prob (F-statistics)	0.000227			
DW statistic	2.251347			

*Source* Computed by Authors using Microfit 5.1

*, **, and *** show 10, 5 and 1% levels of significance respectively

The F-statistic value demonstrates that, at the total level, the tax incentives, trade openness and market size have significant effect on the employment creation (measured by the number of jobs created by FDI) over the study time frame. In the model, the estimation of the R-squared (0.809600) demonstrates that around 80.9% changes in employment creation in the short run are being explained by the independent variables in the study. The DW-statistics estimation of 2.251347 affirms that there is no issue of serial correlation.

The coefficient of tax incentives is positive and statistically significant in the short run, which confirms to the positive relationship acquired over the long run. This implies, over the short run there is a positive link between tax incentives and employment creation. A conceivable clarification of this relationship shows that tax incentives attracts more FDI in the economy resulting in increase in job creation. Accordingly, government of Ghana needs to accomplish more to make an empowering domain for effective tax incentives management. This finding concurs with discoveries of Adurahman and Olumide [49].

Contrary to the long-run results, the outcomes additionally uncover that in Ghana, trade openness negatively affects employment creation over the short run. This shows that an expansion in trade openness by 1% prompts a reduction in employment creation by 81.3% points. This negative relationship contradicts the study expectation. A conceivable clarification of this relationship shows that since imports overwhelm the trade in Ghana, and obviously most imports are consumer goods, this may tend to swarm out domestic productivity and employment generation. Policy-makers who devise trade policies are challenged by ascertaining their fiscal impacts. This negative relationship contradicts with the discoveries of Phillips and Goss [56] and Kudaisi [40].

Finally, the coefficient of market size (GDP) is positive and statistically significant, which confirms to the positive relationship acquired over the long run. This implies, over the long run there is a positive connection between GDP and profitability yet a negative relationship exists between them in the short-run period. It showed that tax incentives have increased the rate of capital accumulation and improved efficiency in capital utilization in foreign investment which is essential for job creation in the communities where such projects exists especially which such projects are into manufacturing. Basnett and Sen [57] suggests that expansion in manufacturing and services had an especially good influence on employment. Overall, GDP growth has a minimal influence on employment in agriculture, but value-added growth in the industry has a rather big impact on employment. The finding concurs with discoveries of Martins [53] and Khan [58].

### Model diagnostics and stability tests

According to Hansen [50], the assessed parameters of a time series data could vary after some time. Thus, it is basic to conduct parameter tests to check for model misspecification that may emerge because of unsteady parameters and in this way prompt bias estimate. Table 11 demonstrates the outcomes for the model diagnostics and goodness of fit.

**Table 11:** Model goodness of fit and model diagnostic tests for employment creation model

Test Statistics	LM Version		F Version	
A: Serial Correlation	CHSQ (1) =	.40044 (.841)	F (1, 32)	.30539 [.862]
B: Functional Form	CHSQ (1) =	.2643 (.607)	F (1, 32)	.20263 [.656]
C: Normality	CHSQ (2) =	29.023 (.000)	Not applicable	
*D: Heteroscedasticity	CHSQ (1) =	.27887 [.597]	F (1, 40)	.26737 [.608]

*Source* Computed by the Authors using Microfit 5.1

Also, Table 11 shows that the errors are normally distributed and the model passes the Ramsey’s RESET for correct specification of the model as well as the white heteroskedasticity test.

Finally, to check the stability of the coefficients of the model, the study employed the CUSUM and CUSUMSQ of recursive residuals stability tests as recommended by Pesaran and Pesaran [51]. According to Bahmani-Oskooee [52], the null hypothesis for this test is that the coefficient vector is the same in every period. The CUSUM and CUSUMSQ of recursive residual stability test in Figs. 2 and 3 indicate that all the coefficients of the estimated model are stable over the study period since they are within the 5% critical bounds.

**Fig. 6:**
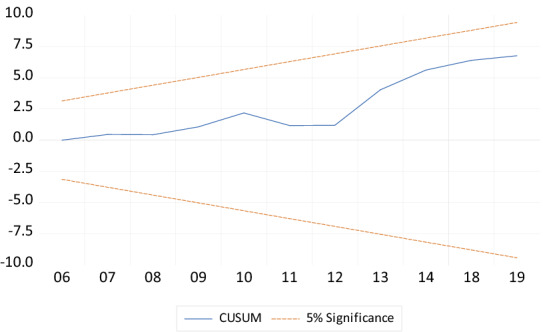
Plot of cumulative sum of recursive. *Source* Generated by the author using Microfit 4.1

**Fig. 7:**
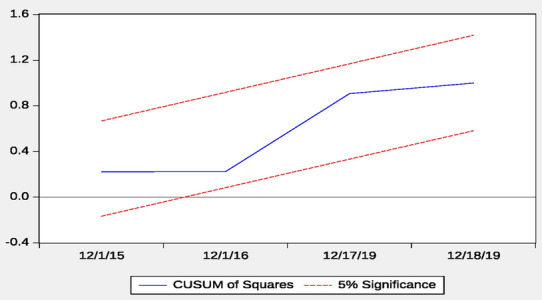
Plot of cumulative sum of squares of recursive residuals. *Source* Generated by the author using Microfit 4.1

From Figs. 4 and 5, the variable on the vertical axis is residuals whiles the variable on the horizontal axis is years. The variable on the vertical axis is residuals whiles the variable on the horizontal axis is years. The CUSUM and CUSUMSQ graph indicates that the coefficients in the models are stable over the study period (Figs. 6, 7).

### The relationship between location tax incentives and the regional distribution of FDI before and after the introduction of the incentive system in Ghana

The government hoped that by establishing a location tax incentive, regions with low corporate taxation rates would attract more FDI. Correlation analysis was used in the study to determine the relationship between location tax incentives and FDIs.

The government of Ghana's approach to giving the location tax incentive was to impact the area of FDI away from the Gt. Accra regions to different regions, particularly, to the three northern regions of the Northern region, the Upper West Regions, and the Upper East Regions. The outcome demonstrates that Gt. Accra has a higher taxation rate, but it draws in more FDI than different regions that have a lower rate. Table 9 establishes no significant relationship between location tax incentives and FDI projects as indicated by (*r* = 0.126, *P* > 0.05). It reveals that there is no connection between location tax incentives and regional distribution of FDI in Ghana. This implies location tax incentives have not conveyed projects to the less endowed regions of Ghana. In quest for this vision, and as far back as 2004, location tax incentives in the structure decreased corporate tax for agro-preparing firms and firms that produce cocoa based items from cocoa waste, it was reported. The package included a 20% corporate tax for firms located in Accra and Tema, a 10% corporate tax for firms located in the other regional capitals, excluding Tamale, Wa, and Bolga, and no corporate tax for firms located in the Northern, Upper East, and Upper West regions. The general corporate tax rate was reduced to 25% in 2006.organizations that are situated in Accra and Tema pulled in at a similar pace of 25%, while firms that are situated in other regional capitals drew in 18.75%. Firms located outside of regional capitals earn 12.50%. Within the period, the regions have gotten a serious and generous measure of FDI, yet it is not yet realized whether the location tax incentive has had any impact on the local appropriation of FDI in the nation. The findings so far confirm the findings of ActionAid Ghana [9] and Van Parys and James [59] that location incentives are ineffective policy tools to influence the location decisions of FDI but contradict the findings of Iwasaki and Suganuma [60] for Russia. Van Parys and James [59] suggest that countries with weak investment climates must concentrate on improving their investment climates instead of reducing taxes (Table 12).

**Table 12:** The relationship between location tax incentives and FDI correlations

				Location tax incentives	FDIs projects
Location Tax Incentives	Pearson Correlation			1	−.126
	Sig. (2-tailed)				.730
	*N*			10	10
	Bootstrap^c^	Bias		0	−.064
		Std. Error		0	.305
		95% Confidence Interval	Lower	1	−.696
			Upper	1	.653
FDI projects	Pearson Correlation			−.126	1
	Sig. (2-tailed)			.730	
	*N*			10	10
	Bootstrap^c^	Bias		−.064	0
		Std. Error		.305	0
		95% Confidence Interval	Lower	−.696	1
			Upper	.653	1

*Source* Author’s Construct, 2022

^c^Unless otherwise noted, bootstrap results are based on 1000 bootstrap samples

## Conclusion

The Ghanaian government has devised tax regulations bestowing incentives on enterprises and individuals with the intention of conducting business in the nation in order to promote the Ghanaian economy. It has been shown that without adequate tax incentives, no corporation can effectively achieve the goals for which it was founded, particularly at the sustaining stage, given the astronomical cost of manufacturing in Ghana and the attendant challenges with infrastructure facilities. This investigation has gone to great lengths to show that tax incentives have significant effect on community development as well as job creation. It is no longer surprising that the government has acknowledged the significant role tax incentives play in job generation and rural economic growth.

From the study, it been established that appropriate tax incentives are beneficial to companies and encourage them to make investment decisions. More importantly, location tax benefits operate as a motivator for investors by minimizing the impact of local manufacturing and increasing the overall impact of industrialization, which creates jobs and improves rural economic development.

Trade openness and market size significantly affect community development and job creation in Ghana. These are nevertheless consistent with contemporary development and, in general, lead to realistic economic growth. Despite the location tax benefits provided by the government during the 1990s, the results show that FDI appropriation is biased towards Greater Accra, Ashanti, Central, Eastern, and Western regions. The study suggests that a more widespread mindfulness campaign, including workshops, courses, and advertisements, be launched to educate investors about available business opportunities, particularly since a significant number of people are wandering the streets due to a lack of employment. They may be able to encourage them to share their knowledge. The government should create tax incentives that emphasize the use of locally sourced goods such as raw materials, labor, and locally manufactured equipment and machinery. As a result, rural development and employment will improve.

## Data Availability

Data and materials are available.

## Abbreviations

CD: Community development
EZ: Economic zone
FDI: Foreign direct investment
GIPC: Ghana Investment Promotion
JSB: Job market
MS: Market size
Top: Trade openness
GDP: Gross domestic product
OLI: Ownership, location and internationalization
OLS: Ordinary least squares
UNCTAD: United Nations Conference on Trade and Development
US: United States
VIF: Variance inflation factors

## References

[1] Tuomia K (2012) Review of investment incentives: bests practice in attracting investement, International Growth Center. Working Paper

[2] Klemm A (2009) Causes, benefits, and risks of business tax incentives. IMF Working Paper, WP/09/21

[3] Francis N (2015) GASB 77: reporting rules on tax abatements. economic development strategies information brief 1. Washington, DC: Urban Institute

[4] Pew Charitable Trusts (2015) Tax incentive programs: evaluate today, improve tomorrow. Economic Development Tax Incentives. Washington, DC: Pew Charitable Trusts

[5] Dunning JH (1997). The European internal market programme and inbound foreign direct investment. J Common Market Stud.

[6] Fujita M, Krugman P, Venables AJ (1999) The spatial economy. Cities, Regions, and International Trade, The MIT Press chap. 5 and 14–19

[7] He C (2002). Information costs, agglomeration economies and the location direct investment in China. Reg Stud.

[8] UNCTAD (2000). Tax incentives and Foreign direct investment: a global survey.

[9] Action aid International (2015) The West African giveaway: use and abuse of corporate tax incentives in ECOWAS. South Africa: Actionaid.org

[10] Institute of Economic Affairs (2012) Tax incentives and exemption regime in Kenya: Is It Working? Issue No. 30

[11] Ghana investment promotion centre (2013) Act 865, Ghana investment promotion centre, Accra

[12] Ministry of finance and economic planning (2004) Budget statement and economic policy of the government of Ghana for the 2014 financial year. Ministry of finance and economic planning, Accra

[13] McDonald I (1995). Decade of enterprise in the United Kingdom. Forum for Applied Research and Public Policy, Winter, 122–125

[14] HUD (1997) State enterprise zone update. U.S. Department of Housing and Urban Development, vol.II., Washington D.C

[15] Bartik TJ, Bingham R (1995) Can economic development programs be evaluated. W.E. Upjohn Institute for Employment Research, Kalamazoo, MI: Staff Working Paper 95–29

[16] Buss TF (2001). The effect of state tax incentives on economic growth and firm location decisions: an overview of the literature. Econ Dev Q.

[17] Boarnet MG (2001). Enterprise zones and job creation: linking evaluation and practice. Econ Dev Q.

[18] Greenbaum R, Engberg J (2000). An evaluation of state enterprise zone policies. Policy Stud Rev.

[19] Rousseau PL, Wachtel P (2005) Economic growth and financial depth: Is the relation extinct already? UNU/WIDER conference on Financial Sector Development for Growth and Poverty Reduction, July, Helsinki

[20] World Bank (2021). Ghana rising – accelerating economic transformation and creating jobs.

[21] Raifu IA, Aminu A, Folawewo AO (2020). Investigating the relationship between changes in oil prices and unemployment rate in Nigeria: linear and nonlinear autoregressive distributed lag approaches. Futur Bus J.

[22] Obiakor RT, Okere KI, Muoneke OB (2022). Accounting for the symmetric and asymmetric effects of FDI-growth nexus amidst financial crises, economic crises and COVID-19 pandemic: application of hidden co-integration. Futur Bus J.

[23] Okere KI, Muoneke OB, Onuoha FC (2022). Tripartite relationship between FDI, trade openness and economic growth amidst global economic crisis in Nigeria: application of combined cointegration and augmented ARDL analysis. Futur Bus J.

[24] Dada JT, Akinlo T (2021). Foreign direct investment and poverty reduction in sub-Saharan Africa: Does environmental degradation matter?. Futur Bus J.

[25] Kirikkaleli D, Adeshola I, Adebayo TS (2021). Do foreign aid triggers economic growth in Chad? A time series analysis. Futur Bus J.

[26] Leedy PD (1993). Practical research: planning and design.

[27] Panneerselvam R (2006). Research methodology.

[28] Cleeve E (2008). How effective are fiscal incentives to attract FDI to Sub-Saharan Africa?. J Dev Areas.

[29] Fowowe B (2013). Do Fiscal incentives promote investment?: empirical evidence from Nigeria. The J Dev Areas.

[30] Agyei KN, Amankwaah E (2018). Trade tax revenue and trade openness in Ghana. J Emerg Trends Econ Manag Sci (JETEMS).

[31] Adam C, Tram N, Pranka C, Schildt C, Sheu J and Whitcomb ER (2011). Job creation: a review of policies and strategies. IRLE Working Paper No. 105–11.http://irle.berkeley.edu/workingpapers/105-11.pdf

[32] Anyanwu J (2011) Determinants of FDI flows to Africa, 1980–2007. Tunis: African Development Bank Group, Working Paper Series No. 136, Tunisia

[33] Bertheau RLI (2012) Geographic, technological and economic analysis of isolated diesel grids; Assess- ment of the upgrading potential with renewable energies for the examples of Peru, the Philippines and Tanzania. www.reiner-lemoine-institut.de/sites/default/files/bertheau2012_paper_geographic_techno-logical_and_economic_analysis_of_isolated_diesel_grids_5thired_berlin.pdf

[34] Naudé WA, Krugell WF (2007). Investigating geography and institutions as determinants of foreign direct investment in Africa using panel data. Appl Econ.

[35] Cevis I, Burak C (2007) The economic determinants of foreign direct investment in developing Countries and transition economies. The Pakistan Development Review, pp 285–299

[36] Davidson R, MacKinnon J (1993). Estimation and inference in econometrics.

[37] Pesaran MH and Shin Y (1999) An autoregressive distributed lag modelling approach to co- integration analysis, in Econometrics and Economic Theory in the 20th Century: the Ragnar Frisch Centennial Symposium, eds. Storm, S., Cambridge University Press, Chapter 11, pp1–31

[38] Pesaran MH, Shin Y, Smith RJ (2001). Bounds testing approaches to the analysis of level relationships. J Appl Economet.

[39] Charemza WW and Deadman DF (1992) New directions in econometric practice: general to specific modelling, Cointegration and Vector Autoregression, Edward Elgar, Aldershot

[40] Kudaisi BV (2014). An empirical determination of FDI in west Africa countries: a panel data analysis. Int J Dev Econ Sustain.

[41] El Ha H, Zenjari A and Mostapha C (2012) The impacts of taxation on investment decisions: the case of Morocco. National School of Business and Management, Hassan the 1st University, Settat, Morocco

[42] Fahmi MR (2012) Analyzing the relationship between tax holiday and foreign direct investment in Indonesia. Asia pacific studies ritsumeikanasia pacific university Japan

[43] Stapper M (2010) Tax regimes in emerging Africa: Can corporate tax rates boost FDI in sub-Sahara Africa?, ASC Working Paper 88 / 2010, African Studies Centre Leiden, The Netherlands

[44] Simovic H, Zaja M (2010). Tax incentives in Western Balkan countries. World Academy of Science, Engineering and Technology. Int J Soc Bus Psychol Hum Sci Eng.

[45] Etim RS, Jeremiah MS, Jeremiah OO (2019). Attracting foreign direct investment (FDI) in Nigeria through effective tax policy incentives. Int J Appl Econ Financ Acc.

[46] Banerjee JD, Mestre R (1998). *Error Correction* mechanism tests for cointegration in a single-equation framework. J Time.

[47] Bond WJ, Woodward FI, Midgley GF (2005). The global distribution of ecosystems in a world without fire. New Phytol.

[48] Abdurahman E and Olumide B (2021) Implications of tax incentives on foreign direct investment decisions. Taxaide Professional Services, Lagos State

[49] Kondo K and Okubo T (2020) The impact of market size on firm selection, discussion papers 20053, Research Institute of Economy, Trade and Industry (RIETI)

[50] Hassan I, Koetter M, Lensik R and Meesters AJ (2008) Bank efficiency, financial depth, and economic growth. University of Groningen, Faculty of Economics & Business and CIBIF, Netherlands

[51] Pesaran MH and Pesaran B (1997) Working with Microfit 4.0 Interactive Econometric Analysis; Windows Version. Oxford University Press, Oxford

[52] Bahmani-Oskooee M, Kantipong T (2001). Bilateral J-curve between Thailand and her trading partners. J Econ Dev.

[53] Martins P (2013) Growth, employment and poverty in Africa: tales of lions and cheetahs. Background paper. Washington D.C.: World Development Report 2013

[54] Hussain, Kabibi KF (2012) Determinants of foreign direct investment flows to developing countries. SBP Res Bull 8(1):23–45

[55] De-Graft AH and Acheampong L (2017) Comparing parametric and semiparametric error correction models for estimation of Long run equilibrium between exports and imports, APSTRACT: applied studies in agribusiness and commerce, AGRIMBA, vol 11(1–2), September

[56] Phillips JM, Goss EP (1995). The effect of state and local *taxes* on economic development. South Econ Rev.

[57] Basnett Y, Sen R (2013) What do empirical studies say about economic growth and job creation in developing countries? overseas Development Institute (oDI), London. Retrieved from https://assets.publishing.service.gov.uk/media/57a08a2340f0b652dd0005a6/Growth_and_labour_absorption.pdf. Accessed 6 Dec 2021

[58] Khan AR (2007) Growth, employment and poverty: an analysis of the vital nexus based on some recent UNDP and ILO/SIDA studies, U.N. DESA Working Paper No. 49. New York: The United Nations

[59] Van Parys S, James S (2010). The effectiveness of tax incentives in attracting investment: the case of the CFA franc zone.

[60] Iwasaki I, Suganuma K (2005). Regional distribution of foreign direct investment in Russia. Post-Communist Econ.

